# The Validation of the Decisional and Emotional Forgiveness Scale among a Chinese Sample: The Mediating Role of Forgiveness between Stress Perception and Resilience

**DOI:** 10.3390/ijerph192316267

**Published:** 2022-12-05

**Authors:** Wenyuan Wang, Suyao Liu, Everett L. Worthington, Haijiang Li

**Affiliations:** 1 Department of Psychology, College of Education, Shanghai Normal University, Shanghai 200234, China; 2 The Research Base of Online Education for Shanghai Middle and Primary Schools, Shanghai 200234, China; 3 Department of Psychology, Virginia Commonwealth University, Richmond, VA 23284, USA

**Keywords:** Chinese sample, decisional forgiveness, emotional forgiveness, validation

## Abstract

This study aimed to revise and test the reliability and validity of the Chinese version of the Decisional and Emotional Forgiveness Scale. In experiment 1, 1171 college students and postgraduates were invited to complete the questionnaire that provides the data for this scale. The results from this, following exploratory factor analysis, showed that the factor loading values met the standards detailed in the past literature, except in the case of item C2. The results of confirmatory factor analysis (while excluding item C2) showed a good structure validity. Furthermore, it also showed that a four-factor model fit the data well and that the reliability values (including internal consistency and test–retest reliability) met the commonly held standards. Decisional and emotional forgiveness subfactors were significantly correlated with transgression-related interpersonal motivations and self-construal. Experiment 2 was conducted in order to further confirm the validity of the scale: the results of mediated analysis showed that emotional forgiveness and the path from decisional forgiveness to emotional forgiveness could mediate the relationship between stress perception and resilience. Thus, the revised Chinese version of the Decisional and Emotional Forgiveness Scale showed good reliability and validity within a Chinese sample, demonstrating its usability as an effective tool to evaluate college students’ level of decisional and emotional forgiveness.

## 1. Introduction

After being offended by an aggressor, individuals may feel angry or hostile, or even decide to take revenge on this aggressor. These destructive reactions usually have a negative psychological and physical influence on individuals. In this context, research shows that: forgiveness plays a key role in restoring damaged relationships and reducing the negative influence of conflicts [1]; negatively correlates with neuroticism [2], depression [3], and rumination [4]; and positively correlates with subjective wellbeing [5] and interpersonal relationships [6]. Forgiveness has also been shown to affect physical health, as individuals with lower levels of forgiveness are more likely to have higher blood pressure, heart rate, and perceived stress [7], as well as more sleep quality problems [8]. These findings suggest the importance of forgiveness for both individuals and society.

Notwithstanding the above, the concept of forgiveness is convoluted. North [9] advised that forgiveness essentially requires one to recognize a wrongdoer’s responsibility for the latter’s action. Further, it typically involves one’s conscious attempt to improve oneself in relation to the wrongdoer. Enright [10] emphasized the reduction in negative cognition, emotion, and behavior; and the increase in positive cognition, emotion, and behavior toward the offender. Meanwhile, McCullough and Michael [11], as well as and McCullough et al. [12] suggested that forgiveness entails a pro-social change in motivation, where there is a decrease in one’s avoidance and revenge motivation and an increase in benevolent motivation toward an offender.

Researchers have also proposed two types of forgiveness: decisional and emotional forgiveness. The first refers to a behavioral intention shift regarding a relationship, going from a greater inclination toward revenge-related and avoidance-related behaviors to a greater inclination toward restoration behaviors [13]. Meanwhile, emotional forgiveness refers to the emotional shift process where one goes from negative and unforgiving emotions into positive and other such oriented emotions toward an offender [6,14]. On the topic, in research, it has been remarked that individuals may decide to forgive and to not engage in revenge behaviors toward offenders, while maintaining their negative emotions (e.g., anger and resentment) toward them [6,14,15].

In order to scientifically measure these two types of forgiveness, researchers have developed the Decisional and Emotional Forgiveness Scale (DEFS) [16], which has had its validity and reliability evaluated and showed excellent results [17,18,19,20,21]. Although Watkins et al. [18] found that both decisional and emotional forgiveness can negatively predict individuals’ avoidance and revenge motivation, others have showed differences between these two types of forgiveness; for example, Bartholomaeus and Strelan [21] found that decisional forgiveness is associated with an individuals’ beliefs in a just world and in destiny, while emotional forgiveness did not associate with these two. Self-construal is conceptualized as a constellation of thoughts, feelings, and actions concerning one’s relationship to others, and the self as distinct from others [22]. Of particular note, individuals tend to hold interdependent self-construal in collectivist cultures, whereas those in individualist cultures hold independent self-construal [22]. In addition, previous studies have found that individuals from different cultures tend to engage in different forgiveness types. Within collectivist cultures, individuals tended toward decisional forgiveness, whereas this not true for those within individualist cultures; this may be due to the fact that they have a higher willingness to maintain harmony in their interpersonal relationships when compared with people from individualist cultures [17,20,23].

Although the DEFS has been widely used, few studies have used this scale with Chinese (especially in mainland China) samples. For example, while attempting to explore the differences in decisional and emotional forgiveness by individuals’ cultural values (i.e., individualist and collectivist), Hook et al. [20] used the DEFS among Chinese and New Zealand samples. However, they did not conduct a standardized revision for the tool, moreover, there was no relevant reliability and validity indicator. In addition, their results showed that item A4 (i.e., from the decisional forgiveness scale [DFS]) showed a low correlation with other items in the scale, leading to its exclusion from the final analysis [20]. In two other studies conducted with Chinese samples, item C2 (i.e., from the Emotional Forgiveness Scale [EFS]) was removed owing to having a low discriminant validity [24,25]. Thus, the few studies that exist on the topic have presented inconsistent findings, thereby highlighting a research gap and a suggestion on the need to further analyze the psychometric properties of the Chinese version of the DEFS. Accordingly, this study aimed to revise and test the reliability and validity of the Chinese version of the DEFS, as well as its applicability and suitability to Chinese samples. The final goal of this research was to provide an effective measurement tool for decisional and emotional forgiveness in China.

## 2. Study 1

### 2.1. Materials and Methods

#### 2.1.1. Participants

We collected data through two survey waves. Off-line and on-line surveys were conducted for the first survey, but only an online survey was conducted for the second survey due to the COVID-19 pandemic. The participants of the study were composed of undergraduate and graduate students, who were randomly recruited via a blinded for review process. Data were deleted when duplicated submissions were made using a single ID (i.e., used twice or more), as well as participants who did not choose the specified options for additional items (i.e., please choose “strongly disagree”). Finally, in Sample 1, 611 participants were included (238 female; Mage = 20.75, and SD = 2.01), and they completed only the DEFS [16]; their data were used for the purpose of exploratory factor analysis (EFA). In Sample 2, 560 participants were included (387 female, Mage = 18.54, SD = 1.07), where they completed the DEFS and other measures; their data were used for the purposes of confirmatory factor analysis (CFA). For the CFA, Schreiber et al. [26] have suggested that the ratio of sample size to number of items should be ≥ 10:1; accordingly, we calculated that a sample with 400 participants would meet this standard. The study was conducted in accordance with the guidelines detailed in the Declaration of Helsinki and was approved by the Ethics Committee of the School of the Department of Psychology, Shanghai Normal University.

#### 2.1.2. The Chinese Version of the DEFS

Our revised Chinese version of the DEFS included its two original subscales, namely DFS and EFS. The DFS includes the inhibition of harmful intention (IHI) and pro-social intention (PI) factors, while the EFS includes the presence of positive emotions (PE) and the reduction in negative emotions (RE) factors. The questionnaire requested participants to recall a situation where someone had hurt them and to rate their agreement with the description in each item. The items were rated on a 5-point scale, ranging from 1–5 (strongly disagree–strongly agree).

First, we requested expert psychological researchers (with accumulated knowledge on the concept of forgiveness) to translate the DFS and EFS subscales to Chinese. Each sentence was then modified repeatedly in order to form the preliminary questionnaire draft. Then, we invited postgraduate students (who had majored in English) to conduct backwards translation and to remodify the scale according to the translation results. Finally, we invited a psychology professor to check the questionnaire—specifically to suggest modifications to make the language expressions in the items as fluent and clear as possible—as well as to ensure that this revised Chinese version of the DEFS was not substantially different from the original.

#### 2.1.3. Transgression-Related Interpersonal Motivations Inventory

We used the 18-item Transgression-Related Interpersonal Motivations Inventory (TRIM-18) [27] in order to measure forgiveness. It possesses 3 subscales: revenge motivation (which includes 5 items, e.g., “I want to see him/her hurt and miserable”); avoidance motivation (7 items, e.g., “I am living as if he/she does not exist, is not around”); and benevolence motivation (6 items, e.g., “I have given up my hurt and resentment”). Each item requested for the participants to recall a situation where someone had hurt them and to rate their agreement with the description in the item. Moreover, the items were rated on a 5-point scale, ranging from 1–5 (strongly disagree–strongly agree). In this study, the Cronbach’s α of the subscales were 0.89 for revenge motivation, 0.87 for avoidance motivation, and 0.78 for the benevolence motivation.

Based on previous findings, the TRIM-18 is excellent for measuring interpersonal forgiveness levels [1,27]. In addition, cross-cultural research has suggested that individuals living in collectivist nations are more likely to forgive past offenders [18,20]. Although there is a plethora of research on interpersonal forgiveness when comparing differences across nations, fewer scholars have attempted to examine differences between individuals within a nation. We thus attempted to fill in this research gap, and we used this measurement tool for checking the convergent validity of our revised DEFS.

#### 2.1.4. Self-Construal Scale

We utilized the 24-item Self-construal Scale (SCS) [22] in order to measure self-construal. It has two subscales: independent self-construal (which includes 12 items, an example item of which is “Speaking up during a class is not a problem for me”) and interdependent self-construal (12 items; an example item is “My happiness depends on the happiness of those around me”). Both of these sub-scale required that the participants responded on a 7-point scale, ranging from −3–3 (strongly disagree–strongly agree). In the original study, the Cronbach’s α of the subscales were 0.69 and 0.73 for the independent and interdependent self-construal, respectively [22]. However, in our study, they were 0.75 and 0.82, respectively.

As China is a nation deemed to be more collectivist than New Zealand (as well as many other Western nations [20]), we chose to collect participants’ self-construal levels in order to confirm whether their forgiveness was more strongly associated with decisional forgiveness than with emotional forgiveness.

#### 2.1.5. Data Analysis

The descriptive statistics (i.e., t-test, internal consistency coefficients, and convergent validity) was conducted using SPSS, version 23.0. The Kaiser–Meyer–Olkin (KMO) test and Bartlett’s spherical tests were conducted in order to ensure sample suitability for factorial analysis before conducting EFA. In order to verify the structural model of the DEFS, EFA and CFA were conducted using a weighted least squares, mean, and variance adjusted (WLSMV) estimator in order to treat the ordinal data by MPlus Version 7.4 [28]. As all factors were assumed to be related based on previous studies, an oblique (Geomin) rotation was performed [29,30]. The following indices, which were deemed as effective and reliable indicators by prior research [26,31], were used to confirm the model: comparative fit index (CFI) and the Tucker–Lewis index (TLI), which must each be higher than 0.90; as well as the root-mean-square error of approximation (RMSEA), which is acceptable when lower than 0.08 [32]. In addition, the theoretical four-factor structure was imposed to investigate the hypothesized structure of the DEFS. Furthermore, age and gender were treated as covariates.

### 2.2. Results

#### 2.2.1. EFA

Before examining the correlation between the variables, we conducted KMO and Bartlett’s sphericity tests. The results showed that Sample 1 was suitable for factorial analysis (KMO = 0.81, Bartlett, *χ*^2^ = 3242.67, and *p* < 0.001).

The results showed that a five-factor model had eigenvalues greater than 1.00; moreover, this model was inconsistent with that of the original scale. After checking the results, we found that item C2 (“I feel sympathy toward him or her”) showed a Geomin factor loading in the original subscales of less than 0.32 [33]; consequently, C2 needed to be removed.

Therefore, using the same procedures, we conducted EFA without item C2. The KMO and Bartlett’s sphericity tests were conducted again. Following this, Sample 1 again showed suitability for factorial analysis (KMO = 0.81, Bartlett, *χ*^2^ = 3146.46, and *p* < 0.001). Now, the results showed that a four-factor model had eigenvalues greater than 1.00; this was consistent with the model of the original scale; in addition, all indices met the required standard (RMSEA = 0.04, CFI = 0.98, and TLI = 0.96). The Geomin factor loading of the items ranged from 0.40–0.95 (see Table 1).

#### 2.2.2. The Details of the CFA

**Figure 1:**
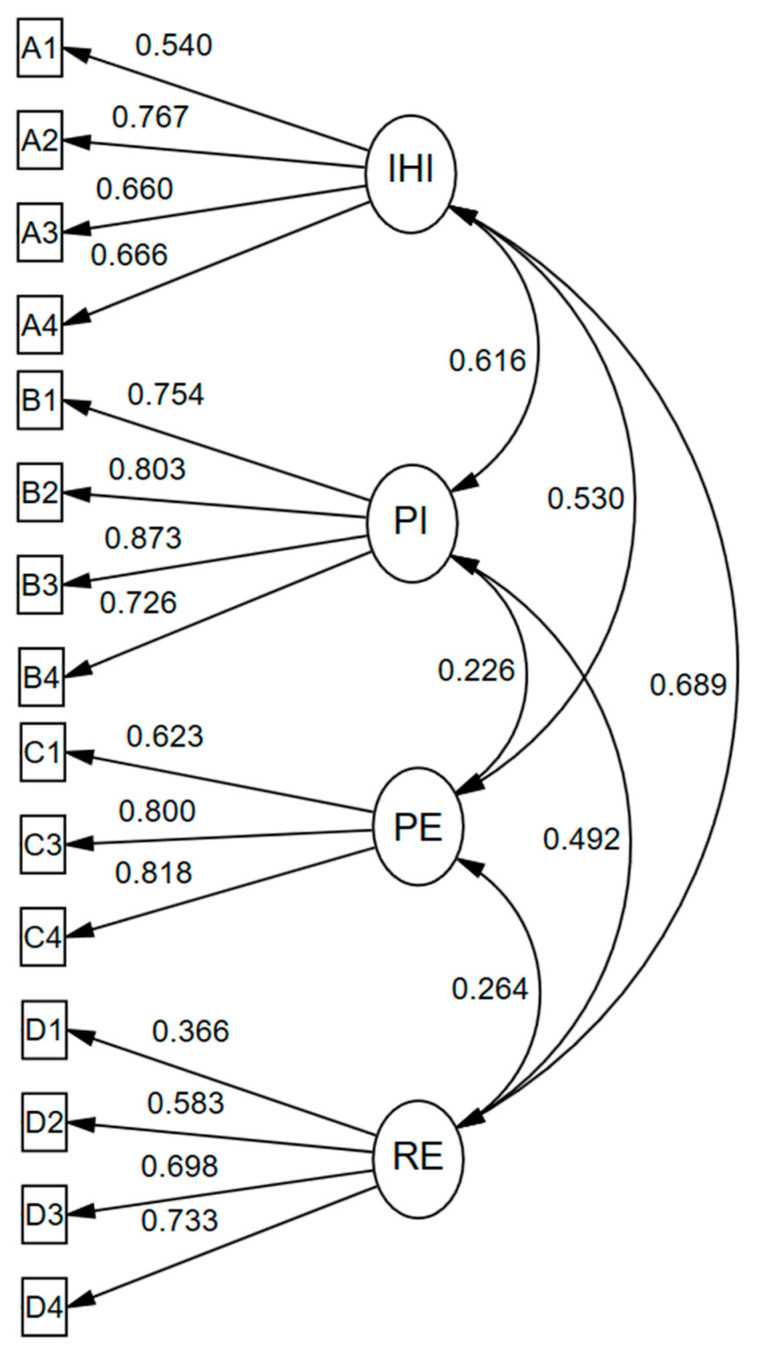
The results of standardized estimates for DEFS structures.

Based on the aforementioned results, we excluded item C2 from the CFA. First, we conducted the KMO and Bartlett’s sphericity tests, which showed that Sample 2 was suitable for factorial analysis (KMO = 0.86, Bartlett, *χ*^2^ = 3372.73, and *p* < 0.001). The CFA results showed that the values for the subscales of the Chinese version of the DEFS that were consistent with those of the original scale (*χ*^2^ = 382.36, *χ*^2^*/df* = 3.86, CFI = 0.91, TLI = 0.89, and RMSEA = 0.07). As the TLI is slightly lower than 0.9, we modified the model according to certain modification indices (MI; [34]). Specifically, item D2 was correlated with item D1, and C1 was related with A4. After the modification, all the indices meet the standard (*χ*^2^ = 321.87, *χ*^2^*/df* = 3.32, CFI = 0.93, TLI = 0.91, and RMSEA = 0.06). The results of the standardized estimates can be seen in Table 2 and Figure 1.

#### 2.2.3. Reliability

We measured the Cronbach’s α (i.e., internal consistency) of each subscale (i.e., DFS and EFS) in both Samples 1 and 2. In addition, we noticed that Cronbach’s α would be higher if item C2 was deleted; accordingly, the results below are those for the questionnaire without item C2. For Sample 1, both subscales and its factors presented results above the standard (DFS, IPI: *α* = 0.80, PI: *α* = 0.70; EFS, PE: *α* = 0.83, and RE: *α* = 0.69), except for the RE dimension, which was slightly below the standard. For Sample 2, the results for all subscales and factors were above the standard (DFS, IPI: α = 0.87, PI: *α* = 0.75; EFS, PE: *α* = 0.78, and RE: *α* = 0.72). The test–retest reliability results demonstrated that the r-value of both subscales met the standard (DFS: *r* = 0.81, *p* < 0.001; EFS: *r* = 0.78, and *p* < 0.001).

#### 2.2.4. Convergent Validity

We selected TRIM-18 and SCS as the criteria. For TRIM-18, the results of Pearson’s correlation analysis showed that the IFI, PI, PE, and RE were all significantly positively correlated with benevolence motivation (*rs* > 0.40, and *p* < 0.001). In addition, these all significantly and negatively correlated with revenge and avoidance motivation (*rs* < −0.3, *p* < 0.001). For the purposes of the SCS, DFS and its subscales were significantly positively correlated with independent and interdependent self-construal; further, EFS and its subscales were significantly positively correlated with interdependent self-construal (see Table 3).

### 2.3. Discussion

This study aimed to revise and test the reliability and validity of the Chinese version of the DEFS and its applicability to Chinese samples. Our results showed that all psychometric indicators, namely EFA, CFA, reliability (internal consistency and test–retest reliability), and convergent validity reached the threshold of standard results, thereby suggesting that our revised version of the DEFS may be an excellent tool for measuring forgiveness in China.

According to the results of the EFA in Sample 1, the factor loading of item C2 for the original dimension was less than 0.32. This is consistent with previous research [24,25], which found that the correlation between item C2 and the score of EFS was relatively low. However, Hook et al. [20] found that participants from China had a different understanding of item A3, while those from New Zealand had a different understanding of item C2. In our study, the EFA results suggested that item A3 was suitable for our Chinese sample. Thus, we deleted item C2 and conducted EFA; after this deletion, our revised Chinese version of the DEFS presented a relatively stable four-factor structure and factor loadings greater than 0.4. In addition, after deleting item C2, the results of CFA showed that our revised Chinese version of the DEFS excellently fit the four-structure model of the original scale, thereby showing an RMSEA value lower than the standard. According to the fit indices of this model, our revised DEFS conformed to statistical standards and was consistent with the theoretical constructs it measures. Regarding reliability, the internal consistency and test–retest reliability of the revised DFS and EFS subscales, as well as their factors, were all above 0.7, except for the RE factor in Sample 1. Therefore, our revised Chinese version of the DEFS had high internal consistency, internal logic, and adequate test–retest reliability.

Regarding convergent validity, Watkins et al. [18] found that both the DFS and EFS subscales were negatively correlated with the avoidance and revenge motivations. Consistent with these findings, Bartholomaeus and Strelan [21] found that both DEFS subscales were positively correlated with benevolence motivation. Thus, we selected the TRIM-18 in order to confirm the convergent validity of our revised Chinese version of the DEFS. Both DFS and EFS and their subscales were found to be negatively correlated with revenge and avoidance motivation and positively related to benevolence motivation. In addition, many cross-cultural studies have been conducted in order to explore differences in forgiveness across nations, with most finding that those who come from collectivist nations tend to forgive offenders [20,23,31]. The present study confirmed that opinion again. However, although decisional and emotional forgiveness correlated with interdependent self-construal, the correlation between independent self-construal and emotional forgiveness did not reach the threshold of significance.

In order to further confirm the validity of the revised scale, we conducted Study 2 to test the relationship between stress perception, emotional forgiveness, decisional forgiveness, and resilience. According to the stress and coping model, individuals may use specific coping strategies in specific contexts when they perceive stress [35]. Researchers have advised that forgiveness as a coping method may play a critical role in the stress and coping model [36,37]. Chi et al. [24] found that benign attributions and empathy could facilitate a higher level of decisional and emotional forgiveness. In addition, emotional forgiveness and the path from decisional forgiveness to emotional forgiveness could mediate the relationship between gratitude and satisfaction in romantic relationships [25]. That is to say, both decisional forgiveness and emotional forgiveness were potential strategies when faced with stress. Moreover, resilience as an critical psychological resource [38] is negatively related to stress perception [39]. The previous study indicated that forgiveness as a coping strategy could significantly promote resilience. Specifically, forgiveness can promote resilience by improving mental health, dislodging individuals from rumination, and focusing on others rather than on themselves [40].

Although previous studies have confirmed the relationship between forgiveness, stress perception, and resilience, the roles of decisional forgiveness and emotional forgiveness are still unclear. Moreover, decisional forgiveness appears to be a useful strategy for promoting emotional forgiveness. This is due to the fact that making a decision to forgive others means that individuals deliberately regulate negative emotions and usually achieve emotional forgiveness [17,18]. Therefore, the present study proposed that resilience may bounce back well if individuals decide to forgive and achieve emotional forgiveness when they faced stress.

## 3. Study 2

### 3.1. Materials and Methods

#### 3.1.1. Participants

Data that included a total of 1101 participants (705 females; Mage = 22.55, SD = 3.49) were obtained via online survey. Data were deleted when duplicated submissions were made using a single ID (i.e., used twice or more), as well as when participants did not choose the specified options for additional items (i.e., please choose “strongly disagree”). The study was conducted in accordance with the guidelines detailed in the Declaration of Helsinki and was approved by the Ethics Committee of the School of the Department of Psychology, Shanghai Normal University.

#### 3.1.2. Perceived Stress Scale (PSS)

We utilized a 14-item Perceived Stress Scale [41,42], which possessed 2 subscales: the positive subscale (which included 7 items, e.g., “how often have you felt unable to control the important things in your life”) and the negative subscale (7 items, e.g., “how often have you dealt successfully with day-to-day problems and annoyances”). Participants were then requested to rate on a 5-point scale, ranging from 0 to 4 (not at all to always) according to the feeling in the last month. In the original study, the Cronbach’s α ranging from 0.78 to 0.91 [42]. In present study, the Cronbach’s α of the subscales were 0.88 and 0.83 for the negative and positive, respectively.

#### 3.1.3. Connor-Davidson Resilience Scale (CD-RISC)

We used the 25-item Chinese version of the CD-RISC [43] in order to measure the ability to cope with stress and adversity. Participants were requested to rate on a 5-point scale, ranging from 0–4 (not true at all–true nearly all of the time). However, different with the original scale, the Chinese version of CD-RISC contains three subscales: tenacity (which includes 13 items, e.g., “You can achieve your goals”), strength (8 items, e.g., “Able to adapt to change”), and optimism (4 items, e.g., “Close and secure relationships”). The higher the total score of the scale, the higher the resilience. For the Chinese version of the CD-RISC, the Cronbach’s α is 0.89 [41,43] and 0.95 in the present study.

#### 3.1.4. Data Analysis

The descriptive and Pearson’s correlation analyses were conducted by using SPSS 23.0. In addition. MPlus (version 7.4) was used to examine the mediation effect of DFS and EFS. Similar to study 1, CFI and TLI (which must each be higher than 0.90) and RMSEA (which is acceptable when lower than 0.08 [32]) were used to test the goodness of fit for the model. A total of 5000 bootstrap samples were used in order to estimate the direct and indirect effects; further, the 95% confidence intervals were bias corrected (CIs) and were then used to test whether the effect is significant (i.e., whether the 95% CI contained zero). Age and gender were treated as covariates. In addition, we also conducted the CFA in order to reconfirm the structure of the revised DEFS, where all indices and the standards were the same as Study 1.

### 3.2. Results

The CFA results showed values for the subscales of the Chinese version of the DEFS that were consistent with those of the original scale (*χ*^2^ = 256.04, *χ*^2^/df = 5.45, CFI = 0.98, TLI = 0.94, and RMSEA = 0.06).

**Figure 2:**
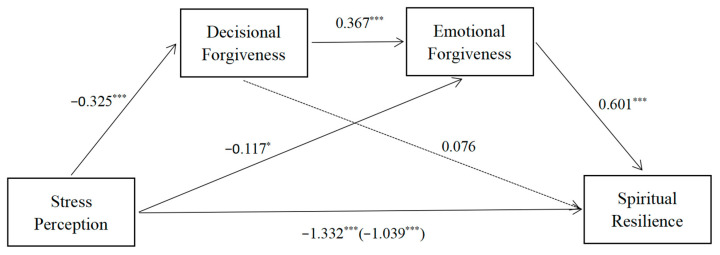
Specific paths and path coefficients for the test model. * *p* < 0.05 and *** *p* < 0.001.

Decisional forgiveness and emotional forgiveness were negatively associated with stress perception and positively associated with resilience. Table 4 displays the Pearson’s correlations among resilience, decisional forgiveness, emotional forgiveness, and stress perception. According to the results of mediation analysis, we found that the hypothesized model was a saturated model (CFI = 1.000, TLI = 1.000, and RMSEA < 0.001). In regard to the direct effect, resilience was negatively associated with stress perception (*β* = −1.039, *p* < 0.001, and 95% CI [−1.153, −0.928]). The first indirect effect was through decisional forgiveness (stress perception → decisional forgiveness → resilience: *β* = 0.025, and 95% CI [−0.085, 0.029]). The second indirect effect was through emotional forgiveness (stress perception → emotional forgiveness → resilience: *β* = −0.070, and 95% CI [−0.107, −0.041]). The third indirect effect was through decisional forgiveness and emotional forgiveness (stress perception → decisional forgiveness → emotional forgiveness → resilience: *β* = 0.072, and 95% CI [−0.101, −0.046]). In other words, resilience was associated with stress perception through decisional forgiveness, emotional forgiveness, and the path from decisional forgiveness to emotional forgiveness. Path coefficients (stress perception → decisional forgiveness: *β* = −0.325,and 95% CI [−0.373, −0.275]; stress perception → emotional forgiveness: *β* = −0.117,and 95% CI [−0.152, −0.081]; decisional forgiveness → emotional forgiveness: *β* = 0.367, and 95% CI [0.322, 0.412]; emotional forgiveness → resilience: *β* = 0.601, and 95% CI [0.395, 0.801]); decisional forgiveness → resilience: *β* = 0.076, and 95% CI [−0.090, 0.254]); stress perception → resilience: *β* = −1.039, and 95% CI [−1.153, −0.928]) (See Figure 2 for more detail).

### 3.3. Discussion

The present study aimed to examine the relationships among stress perception, decisional forgiveness, emotional forgiveness, and resilience, in addition to providing further evidence for the validity of DEFS. We found that decisional forgiveness could not mediate the relationship between stress perception and resilience, but emotional forgiveness and the path from decisional forgiveness to emotional forgiveness could.

According to the stress and coping model, forgiveness is a valuable strategy when individuals face stress [36,37]. Furthermore, the present study echoes this opinion. In the present study, stress perception was negatively associated with decisional and emotional forgiveness—in other words, when individuals perceive stress, they seem less likely to make decisional forgiveness and emotional forgiveness. However, decisional forgiveness and emotional forgiveness were positively associated with resilience. Forgiveness could reduce depression, anxiety, rumination, and stress [3,11]. This was in addition to the fact that it can increase subjective well-being [5] and interpersonal relationships [6], whereby these positive effects can significantly help individuals bounce back quickly [40].

Moreover, the present study also found that the path from decisional forgiveness to emotional forgiveness could mediate the relationship between stress perception and resilience. According to previous studies, making a decision to forgive constantly means that victims deliberately regulate negative emotions. Furthermore, this can usually lead to emotional forgiveness [15,18]. Moreover, the prominent intervention models consider decisional forgiveness as an early step in promoting forgiveness, whereby a large number of studies have confirmed that decisional forgiveness is a significantly useful strategy that can be used to achieve emotional forgiveness [6,17].

In the present study, both decisional forgiveness and emotional forgiveness were negatively associated with stress perception and positively related to resilience, but emotional forgiveness could mediate the relationship between stress perception and resilience rather than decisional forgiveness. That is to say when individuals face stressful events, resilience cannot bounce back through decisional forgiveness alone. As seen in previous studies, forgiveness can help individuals improve mental health, reduce rumination, and focus on others, which promotes resilience [40]. However, although individuals are committed to regulating negative emotions related to stressful events, they may continue to ruminate on stressful events and maintain negative emotions [14,15]. Compared to decisional forgiveness, emotional forgiveness had a more direct effect on physical and mental health [16]. Chi et al. [24] found that the strength of the relationship before transgression could directly predict the levels of emotional forgiveness rather than decisional forgiveness. In addition, it is emotional forgiveness that could mediate the relationship between gratitude and satisfaction in romantic relationships rather than decisional forgiveness [25].

## 4. General Discussion

Previous studies have presented inconsistent findings that used DEFS with Chinese (especially in mainland China) samples. The present study provided evidence for the reliability and validity of the Chinese version of the DEFS. The results of the EFA, the CFA, reliability (internal consistency and test–retest reliability), and convergent validity all reached the required standards. Moreover, in Study 2 it was found that decisional forgiveness and emotional forgiveness play different roles between stress perception and resilience, suggesting that our revised version of the DEFS may be an excellent tool for measuring forgiveness in China.

Regarding convergent validity, TRIM-18 and SCS were selected in order to confirm the convergent validity of the Chinese version of the DEFS. TRIM-18 is thought to be an excellent tool to measure forgiveness [11,27]. In addition, individuals from collectivist nations tend to forgive offenders in order to maintain social harmony, although they concomitantly retain their negative emotions toward offenders [20,23,31]. Nonetheless, a study demonstrated that there are people from collectivist nations who are individuals that engage in emotional forgiveness after a conflict [17]. The present study, which also comprised a sample from China (i.e., a collectivist nation), replicated the previous results. Although the correlation between interdependent self-construal and decisional forgiveness was strong, both decisional and emotional forgiveness correlated with both subscales of self-construal. That is, individuals living in collectivist nations may not only engage in decisional forgiveness but also in emotional forgiveness after they face conflicts. Notwithstanding this fact, we did not collect data from samples in or from individualist nations, and as such, the aforementioned explanations will need to be interpreted with caution. Overall, our research findings demonstrated an excellent convergent validity for our revised scale.

Forgiveness as a useful strategy [36,37] can promote mental health, such as in the case of depression [3], rumination [27], subjective wellbeing [5], and interpersonal relationships [16]. The present study provided preliminary evidence confirming that decisional forgiveness and emotional forgiveness are also useful strategies to help individuals bounce back when faced with stress, although decisional and emotional forgiveness played different roles in the relationship between stress perception and resilience. Although individuals made a decision to forgive, they may still hold negative emotions, such as anxiety and depression [14,15]. Consequently, the influence of decisional forgiveness on resilience may be relatively weak, but emotional forgiveness had a more direct effect on mental health [16]. However, although we found that decisional forgiveness and emotional forgiveness can mediate stress perception and resilience in Study 2, longitudinal research was not conducted. Therefore, the study design did not allow us to investigate the causal direction of these associations. In addition, the present study only collected the data from the college and graduate student sample, where the findings and whether they can generalized to another group is debatable. Therefore, further studies may be required to conduct a longitudinal design in order to measure the mediation effect of decisional forgiveness and emotional forgiveness over time, or to conduct an experimental design in order to establish a causal direction for relationships between stress perception and resilience among different groups.

## 5. Conclusions

Forgiveness plays an important role in reducing rumination, depression, and anxiety, and helping individuals bounce back [40]. Our findings for the revised Chinese version of the DEFS showed that it reached the required standard values across all its psychometric properties, thus potentially serving as an excellent tool for measuring forgiveness in the context of Chinese samples. We hope that our discussions and that this revised scale enables future researchers to more accurately assess this construct and to produce new perspectives that enrich the literature on forgiveness in China.

## Figures and Tables

**Table 1:** Factor loading, eigenvalue and variance explained for the Chinese version of the DEFS.

Items	IHI	PI	PE	RE
A1	0.48			
A2	0.71			
A3	0.66			
A4	0.41			
B1		0.71		
B2		0.84		
B3		0.78		
B4		0.59		
C1			0.50	
C2			<0.32	
C3			0.95	
C4			0.83	
D1				0.40
D2				0.59
D3				0.72
D4				0.62

**Table 2:** The results of standardized estimates for DEFS structures.

Dimensions	Estimate	Standard Error	*p*-Value
IHI-A1	0.540	0.035	<0.001
IHI-A2	0.767	0.024	<0.001
IHI-A3	0.660	0.029	<0.001
IHI-A4	0.666	0.029	<0.001
PI-B1	0.754	0.022	<0.001
PI-B2	0.803	0.019	<0.001
PI-B3	0.873	0.016	<0.001
PI-B4	0.726	0.024	<0.001
PE-C1	0.623	0.031	<0.001
PE-C3	0.800	0.025	<0.001
PE-C4	0.818	0.024	<0.001
RE-D1	0.366	0.044	<0.001
RE-D2	0.583	0.036	<0.001
RE-D3	0.698	0.033	<0.001
RE-D4	0.733	0.032	<0.001
PI-IHI	0.616	0.036	<0.001
PE-IHI	0.530	0.042	<0.001
PE-PI	0.226	0.048	<0.001
RE-IHI	0.689	0.038	<0.001
RE-PI	0.492	0.044	<0.001
RE-PE	0.264	0.053	<0.001

**Table 3:** Correlations between DFS and EFS, with TRIM-18 and SCS.

	Revenge	Avoidance	Benevolence	IT	IN
DFS	−0.788 ***	−0.552 ***	0.625 ***	0.433 ***	0.256 ***
EFS	−0.543 ***	−0.670 ***	0.645 ***	0.206 ***	0.080
PI	−0.586 ***	−0.648 ***	0.672 ***	0.340 ***	0.238 ***
IHI	−0.778 ***	−0.306 ***	0.408 ***	0.409 ***	0.205 ***
PE	−0.330 ***	−0.550 ***	0.543 ***	0.147 ***	0.002
RE	−0.467 ***	−0.453 ***	0.425 ***	0.158 ***	0.110 **

** *p* < 0.01 and *** *p* < 0.001.

**Table 4:** Correlations among CD-RISC, PSS, DFS and EFS.

	1	2	3	4
RISC	-			
PSS	−0.571 ***	-		
DFS	0.319 ***	−0.418 ***	-	
EFS	0.398 ***	−0.398 ***	0.530 ***	-
mean	67.976	24.794	27.244	20.300
SD	16.027	7.362	5.656	4.614

*** *p* < 0.001.

## Data Availability

All requests for raw and analyzed data and materials are reviewed by the author in order to verify if the request is subject to any intellectual property or confidentiality obligations. The data are available from the corresponding author upon reasonable request.
